# “Why won’t they just vaccinate?” Horse owner risk perception and uptake of the Hendra virus vaccine

**DOI:** 10.1186/s12917-017-1006-7

**Published:** 2017-04-13

**Authors:** J. Manyweathers, H. Field, N. Longnecker, K. Agho, C. Smith, M. Taylor

**Affiliations:** 1 Centre for Health Research, Western Sydney University, Sydney, Australia; 2 School of Animal Biology, University of Western Australia, PO BOX 7178, Tathra, NSW Australia; 3 EcoHealth Alliance, New York, NY USA; 4 Centre for Science Communication, University of Otago, Dunedin, New Zealand; 5 School of Science and Health, Western Sydney University, Sydney, Australia; 6 Department of Agriculture and Fisheries, Queensland Centre for Emerging Infectious Diseases, Biosecurity Queensland, Coopers Plains, QLD Australia; 7 Department of Psychology, Macquarie University, Sydney, Australia

**Keywords:** Risk mitigation, Biosecurity, Trust, Protection motivation theory, Hendra virus, Vaccination, zoonosis, Vaccine hesitancy

## Abstract

**Background:**

Hendra virus is a paramyxovirus that causes periodic serious disease and fatalities in horses and humans in Australia first identified in 1994. Pteropid bats (commonly known as flying-foxes) are the natural host of the virus, and the putative route of infection in horses is by ingestion or inhalation of material contaminated by flying-fox urine or other bodily fluids. Humans become infected after close contact with infected horses. Horse owners in Australia are encouraged to vaccinate their horses against Hendra virus to reduce the risk of Hendra virus infection, and to prevent potential transmission to humans. After the vaccine was released in 2012, uptake by horse owners was slow, with some estimated 11-17% of horses in Australia vaccinated. This study was commissioned to examine barriers to vaccine uptake and potential drivers to future adoption of vaccination by horse owners.

**Methods:**

This study examined qualitative comments from respondents to an on-line survey, reporting reasons for not vaccinating their horses. The study also investigated scenarios in which respondents felt they might consider vaccinating their horses.

**Results:**

Self-reported barriers to uptake of the Hendra virus vaccine by horse owners (*N* = 150) included concerns about vaccine safety, cost, and effectiveness. Reduction in vaccination costs and perception of immediacy of Hendra virus risk were reported as being likely to change future behaviour. However, the data also indicated that horse owners generally would not reconsider vaccinating their horses if advised by their veterinarian.

**Conclusion:**

While changes to vaccine costs and the availability data supporting vaccine safety and efficacy may encourage more horse owners to vaccinate, this study highlights the importance of protecting the relationship between veterinarians and horse owners within the risk management strategies around Hendra virus. Interactions and trust between veterinarians and animal owners has important implications for management of and communication around Hendra virus and other zoonotic disease outbreaks.

**Electronic supplementary material:**

The online version of this article (doi:10.1186/s12917-017-1006-7) contains supplementary material, which is available to authorized users.

## Background

During infectious disease outbreaks, risk decisions and management by all stakeholders are compelled by temporal, cultural and social factors [1]. For those involved in managing disease outbreaks, it is fundamental to have a clear understanding of all factors at play in people’s adoption and adaption of officially recommended risk management strategies [2]. Research into risk has long been challenging, largely because of how risk is defined [3, 4]. Scientists, researchers and risk analysts, identified here as “salaried experts”, define risk as a consideration of magnitude of the risk event and the probability of its occurrence using a mathematical approach [5, 6]. However, when considering how people respond to risk, it has been suggested that there is little benefit in discussing the risk itself [7]. Instead, the very different concept of risk perception needs to be considered. Risk perception is influenced by various social and psychological factors, including trust [8], worldview ([9]; Manyweathers, Taylor, Longnecker, unpublished observations], emotion and affect [10, 11], and the effect of social and cultural processes [12]*.* Consequently, when examining people’s decisions about health protective behaviours, an appropriate framework should include consideration of these factors.

The Cultural Theory of Risk posits that risk perceptions reflect and maintain an individual’s preferred type of social organisation and cultural way of life [13]. Within the theory, risk perception is further defined by the extent to which an individual associates their identity within a particular social group and the extent to which they accept a formal hierarchical system of rules [13–15]. The Cultural Theory of Risk considers the social environment, the stakeholders and the bases on which decision are made around risk as part of one holistic process [16]. Therefore, actions taken to avoid some dangers and ignore, or not act upon, others are made within the framework of social arrangements, and these decisions and choices of how to live are taken together [16]. Because salaried experts and lay people alike lack complete knowledge and foresight, decisions required to navigate through complex risk situations are made by relying on affective and mental shortcuts, known as heuristic reasoning [17]. This reasoning is aimed at maximising desired future outcomes in risk situations. But rather than these outcomes reflecting a united goal for all stakeholders, the mental and affective shortcuts adopted by those at risk reflect an individual’s worldview, their sense of who should have control and what should matter [16, 18]. Their subsequent actions are directed at protecting and maintaining this position [10]. Consequently, the spectrum of responses by stakeholders towards the risk surrounding emerging disease infections is vast and requires careful consideration.

Hendra virus (family *Paramyxoviridae*, genus *Henipavirus*) causes periodic respiratory and neurological disease and subsequent fatalities in horses and humans. Flying-foxes (family *Pteropodidae*, genus *Pteropus*) are the natural host of the virus [19, 20], with the Spectacled and Black flying-foxes evidently the most competent host species [21, 22]. The virus was first isolated and identified after the death of more than 20 horses and their trainer in 1994. Horses are thought to become infected via ingestion or inhalation of virus from pasture, water or surfaces contaminated with flying-fox excreta, with recent research suggesting that flying-fox urine is the most likely transmission medium [22]. Infection via direct contamination of mucous membranes may also occur [22–24]. Of the seven human cases of Hendra virus infection, four (57%) have been fatal [21]. All human cases arose following close and direct contact with infected horses [25].

Between 1994 and 2010, there were 14 reported Hendra virus events, including the seven human cases. In 2011, however, 18 Hendra virus events involving over 20 horses across an expanded geographic area were reported in a single three-month period [25]. This unprecedented and alarming scenario added impetus and urgency to the development of a vaccine for horses to prevent infection and transmission to humans [25]. In November 2012, a vaccine based on a viral G glycoprotein, required by the virus for binding to host cells was released. Antibodies against this protein were found to neutralise the virus and prevent disease [25]. The vaccine was initially released under a restricted permit use pending full registration with the Australian Pesticides and Veterinary Medicines Association (APVMA), the government body responsible for the registration of all agricultural and veterinary chemicals in Australia. The permit enabled the vaccine to be used under strict conditions, including administration by a trained and accredited veterinary surgeon to horses that were uniquely identified by microchip, the details of which were recorded on a national online registry. Since its release, the permit for Hendra virus vaccination has undergone several amendments and included instructions for six-monthly boosters. In August 2015, the vaccine became fully registered, with 12 monthly boosters approved in May 2016.

Despite recommendation by the main state government agencies responsible for Hendra virus policy - Queensland Department for Agriculture and Fisheries (QDAF) and New South Wales Department of Primary Industries (NSW DPI) - that vaccination is *‘the single most effective way of reducing the risk of Hendra virus infection in horses’* [26], uptake of the vaccine has been slow [27]. Whilst there is no national registry of horses in Australia, and thus the total number of horses is unknown, it is estimated that the majority of horses remain unvaccinated, with vaccine rates estimated to be around 11-17% [28].

Other risk mitigation strategies aim at property management practices, reducing the possibility of contact between horses and flying-foxes. These include covering food and water sources in the paddock, removing horses from pasture when bats are most active and removing access by horses to fruiting and flowering trees [26]. However, research has highlighted the problematic nature of these approaches, with some horse owners reporting that they find the recommended strategies ineffective and impractical [29].

To implement inclusive and engaging risk management strategies, it is necessary to adopt a holistic consideration of people’s perception of risk and the adoption of protective health behaviours in zoonotic disease outbreaks [30–33]. For the successful implementation of effective public health interventions, providing information about protective strategies and even the production of a vaccine, while necessary, are not enough to ensure adoption of protective behaviours [34, 35]. In fact, previous research suggests that the observed non-compliant behaviours of horse owners around Hendra virus and the vaccine release should have been anticipated [17, 30, 36] and could possibly have been avoided [37]. Research into vaccine hesitancy around human vaccines is growing [38, 39] and could provide insight into possible outcomes for vaccine roll out and uptake in animals.

In order to facilitate a deeper understanding of how horse owners perceive the risk of Hendra virus infection in horses and how this affects decisions around adoption of protective behaviours, the *Hendra Virus Infection and Transmission Dynamics – Conversation with Industry* project was launched, commissioned by the intergovernmental National Hendra Virus Taskforce [40]. Funding was provided by Australian State and Federal departments and the taskforce identified priority areas for research. The current study was tasked with three main aims:

1. To identify factors associated with the uptake of protective strategies to reduce the risk of Hendra virus infection.
2. To identify reasons for non-vaccination of horses against Hendra virus.
3. To consider how engagement with horse owners and uptake of management strategies may be improved.

In order to address the first two aims of the project and more fully understand horse owners’ responses to Hendra virus and to the vaccine, a mixed method approach was adopted [41, 42]. A survey containing open ended questions provided qualitative data: self-reported reasons given by horse owners for not vaccinating their horses against Hendra virus. Quantitative data from the survey was also analysed to consider factors involved in potential uptake of risk mitigation strategies. Results from analysis of these data are reported here. Research examining how engagement with horse owners might be improved is reported elsewhere (Manyweathers, Taylor, Longnecker, unpublished observations).

## Methods

An online survey was designed to explore risk mitigation practices, risk perception, and attitudes to Hendra virus risk of horse owners using both open and closed questions. The survey was directed at horse owners who had elected not to vaccinate their horses against Hendra virus and were living geographically close to the location of a previous Hendra virus case. A qualifying question was used to screen respondents in order that only those respondents that had not vaccinated all or any of their horses could proceed with the survey.

The survey comprised 38 questions with an opportunity to leave contact details for a follow up interview in the second phase of the study. Data collected from the latter are reported elsewhere (Manyweathers, Taylor, Longnecker, unpublished observations).

Respondents were asked about their reasons for not vaccinating their horse(s) and situations that might make them reconsider. Questions concerning perceived barriers to uptake of vaccination were also included, using Likert scales for responses.

The survey was developed and administered using the online hosting software SurveyMonkey™, after piloting with three horse owners and four veterinary professionals, recruited through the first author’s veterinary practice. After the piloting process, demographic questions were altered slightly to ensure that all possible variations of horse ownership, management and current vaccination status were included. The survey is included as a Additional file 1. Ethics approval for the study was provided by the Human Research Ethics Committee of the University of Western Sydney (Protocol No. H10643).

The survey link was posted on the Facebook pages of 26 veterinary hospitals in areas where Hendra virus cases had previously been confirmed: far north New South Wales, South East, Central, and Far North Queensland. The survey was open for six weeks, with three postings on the Facebook page, from January 22nd until March 10th 2015. Because of the uncontrolled nature of social media posting, the location of respondents was determined using a question regarding distance to nearest Hendra virus case and confirmed using respondents’ postcode.

Cross posting from veterinary hospital Facebook groups included a dressage club based in an area where a significant number of Hendra virus cases occurred and a Facebook group with an active presence in the discourse around Hendra virus vaccine.

Sample characteristics were determined by simple descriptive statistics. Descriptive analysis was also used to present data highlighting factors that might influence horse owners to reconsider vaccinating their horses against Hendra virus. Data were managed using IBM SPSS V22. Qualitative data were managed using NVivo and were coded to investigate the reasons for non-vaccination. The analysis was undertaken using the following approach modified from Braun and Clarke [41, 43]. Data familiarisation was undertaken by reading survey responses in detail. Initial codes were considered across the set of surveys until no new codes were required to represent responses, then relevant data were collated for each code. The codes were then grouped into potential themes, defined and named. Quotations from the raw data were used to narrate the themes, illustrating the unique characteristics of the respondent’s perceptions. Respondents’ spelling was corrected and minor grammatical adjustments made for ease of reading. Clarifications from the first author are included in bold square brackets.

## Results

### Sample characteristics

In total 210 respondents associated with 17 veterinary practices completed the survey and qualified for analysis by reporting having unvaccinated horses. Approximately half the sample (50%, *n* = 106) were aged 44 or younger. While some respondents (7%, *n* = 15) reported earning their main source of income from horse related business or activity, just over a quarter (23%, *n* = 49) earned some income from their horse involvement. A cross section of sectors of horse involvement (multiple responses allowed) were represented in the responses, with 62% (*n* = 131) involved in the competitive/equestrian sector, 57% (*n* = 120) involved in the recreational sector, and 19% involved in the working/farming/stock horse sector.

### Barriers to uptake of vaccination

Just under three quarters of the sample responded to the question concerning their reason for not vaccinating, or withdrawing from the vaccination program (71%, *n* = 150). Each respondent gave, on average, two to three reasons for not adopting vaccination as a protective strategy. A total of 424 comments were coded to explore the reasons given for non-vaccination or not continuing with the six-monthly booster required to be compliant with the recommended vaccination protocol. Three main themes were identified from the coding structure: ‘attitude towards the vaccine’, ‘attitude towards authorities’, including veterinarians and the pharmaceutical company that produces the vaccine, and ‘risk assessment’. The ‘attitude towards the vaccine’ theme reflected people’s concerns, including the potential for side effects, the protocol at the time of data collection requiring six monthly boosters, and the cost and the perceived safety of the vaccine. The ‘Risk assessment’ theme included evidence of people’s own risk assessment of their horses and property and the risk of vaccinating a horse that was unwell. The theme ‘attitude to authorities’ reflected consideration given by horse owners to the nature of the relationship between them and those in authority and the relationship between authorities. Comments consisted of a mixture of one word answers and more in-depth responses. Table 1 summarises the coding and themes.

**Table 1:** Reasons for non-vaccination or withdrawal from the vaccination protocol

Theme	Code	N	Description	Example Comments
Attitude towards vaccine (*n* = 310)	Side effects	91	Reference to side effects, whether general or specific	The amount of horses either having a reaction or death from vaccination. (R17)
	Cost	39	Reference to the cost of vaccination	The cost of the vaccine. (R7)
	Testing	40	Reference to the need for more testing of the vaccine or the lack of testing	I’m not willing to vaccinate against Hendra until more testing is done on the vaccine (R14) Not enough research and the way it was rushed to market. (R41)
	Safety	20	Reference to the safety of the vaccine	Until it has been proved for 5 years I am not convinced it is safe. (R54)
	Efficacy	13	Reference to the vaccine not working,	Current vaccination is not proved sufficiently (R13)
	Animal use	17	Reference to breeding animals and lack of safety, or issues with exporting vaccinated horses	Have not heard any proof if safe to use in breeding animals (R12)
	Hendra virus vaccine protocol	21	Reference to concern regarding the protocol, number of vaccines required, vet involvement	The number of vaccines required and the frequency of them. (R21)
	Licence	43	Reference to concern regarding the restricted licence for the vaccine	Unregistered Vaccine. (R17)
	Future intention	13	Indication that vaccination maybe reconsidered in the future	If this is sorted in the future I will consider it more readily. (R14)
	Attitude to vaccination	11	Indications of attitudes to vaccination in general or other vaccines for horses	I do however regularly vaccinate for strangles and tetanus which I see as a bigger threat having had one of my horse contract strangles even after vaccinating. (R28)
	Freedom of choice	2	Indication of desire to make own choice about whether to vaccinate	I believe in pro-choice. (R46)
Risk assessment (*n* = 94)	Risk assessment	70	Evidence of risk assessment, weighing up the odds, insights into where respondents feel the risk lies, includes the risk of side effects from the vaccine and the risk of Hendra virus infection	They are not in contact with other horses. (R8) The chance of reaction is higher than the chance of catching Hendra when precautions are taken. (R43)
	Biosecurity	10	Evidence of adoption or consideration of biosecurity practices	Extremely low flying fox/bat population in our area and no contact with horse from neighbouring properties. Removal of all fruiting trees and regular monitoring of blossoming/flower native trees which coincide with paddock rotations. (R34)
	Horse health	14	Reference to underlying horse health issues which precludes the vaccine being able to be used.	One is not vaccinated as she is elderly and the risk of infection and contact with people is very low. (R32)
Attitude towards authorities (*n* = 36)	Authorities	36	Reference to authorities, whether veterinarians or pharmaceutical companies, their behaviour, motivation	Horses have died from this vaccine and the authorities are not acknowledging this fact!! (R142)
Other (*n* = 1)	Other	1	Comments that do not fit in to current categories but still need to be considered.	Availability (work full time during the week and compete full time on the weekends). (R22)

#### Theme 1: Attitude to the vaccine

The majority of coded responses (310 comments) identified an aspect of the vaccine as the driver behind the decision not to vaccinate. Concern regarding side effects of the vaccine was the single most frequently given reason for non-vaccination. Some respondents reported a first-hand experience with a vaccine side effect, while others recorded anecdotal evidence.

*I only vaccinated due to pressures from vets but am now reconsidering due to risk factors and my horse’s reaction to the vaccine each time it was given.* (R23)

*One of my horses died after receiving the vaccine which has been investigated. I've also heard far too many have bad reactions that have left them permanently damaged.* (R45)

*There have been more cases of a reaction to the vaccine than there is of a horse contracting the virus. The reactions I've heard or read about are quite confronting and scary, which made me reconsider.* (R11)

*Concerns from social media with side effects means that at present I have not done our miniature horse.* (R30)

The fact that the vaccine was being used under a restricted permit and was not fully registered at the time of the survey was frequently reported as proof that the vaccine was unsafe and insufficiently tested (43 comments).

*Because the vaccine is not registered, it's unsafe and has been known to cause adverse reactions to horses and in some cases cause death. I would not give my human child a vaccine that puts them at risks like this so I won't do it to me horses!* (R75)

*I believe that the vaccination was rushed to get out onto the market and that we do not know all of the possible side effects. I also believe that if it was safe, we'd have a similar vaccine for humans.* (R24)

The cost of the vaccine was the fourth most frequently given single response (39 comments) but is only mentioned by eight respondents as their sole reason for not vaccinating. The cost was mentioned in parallel with the protocol at the time requiring six monthly boosters and veterinary administration.

*Also the cost to get them all done every six months by our vet.* (R47)

*High cost of repeated treatments required. If it was a one off vaccine it would be easier to wear the once off cost of the vaccine.* (R67)

#### Theme 2: Risk assessment

Respondents’ comments were coded for references to their consideration of the risk to them of Hendra virus infection, or Hendra virus vaccine side effects (80 comments). References to underlying horse health issues as a barrier to vaccination were included (14 comments). Biosecurity measures adopted in place of vaccination were mentioned in 10 responses.

*The virus is a very weak one and strong hygiene practices on the home front can do a lot to mitigate any related risk.* (R144)

*Minute risk of a Hendra incident in my horses as assessed by myself and my vet.* (R141)

*I believe that the infection rate of both horses and humans to be statistically insignificant. The virus has been around for ever, why the panic now? Does someone know something I don't? Awareness and PPE covers risks.* (R132)

*I have two older horses and a mare I plan to breed with. Having heard of several severe reactions in vaccinated horses (particularly those with pre-existing heart and neurological conditions) I have concluded that the risk of death or serious illness from the vaccination is much higher than from Hendra in my area. Particularly, since one of my older horses has neurological and heart conditions, the risk would be too large for her.* (R73)

*… good biosecurity practice with ANY sick animal is a more sensible approach to take.* (R96)

Some responses were indicative of where respondents perceived the risk of Hendra virus infection would most likely come from. This included infection from contact with other horses (nine comments), and unhygienic stabling conditions (two comments). Flying-foxes were mentioned in five comments.

*Because the risk is very small in our area. We are not transporting our horses to events like shows only to mates’ properties for mustering where the risk is also minimal. If one of our horses gets it we will put them down and aim in protecting the others in all means possible.* (R25)

*The risk is so low, and my experience with the conditions I witnessed at the clinic that experienced the outbreak have left me confident that my horses will not be exposed to this virus, and are not going to die. We had horses for a long time without knowing about Hendra, and nothing has changed other than in my opinion, people keeping horses in unsuitable conditions.* (R04)

*Low risk (no large mobs of flying foxes seen), horses do not travel or have contact with other horses.* (R62)

*Not necessary as in the history of this virus not one horse at any event or well cared for establishment has ever had a case that is not transmitted by poor hygiene and unsafe veterinary practices. (R134)*

There was also mention of the fact that Hendra virus has been around for a while and therefore perhaps does not warrant the intense pressure of immediate and extreme action.

*The virus has been around for ever and only come to light 20 years ago. (R64)*

*Hendra virus is hard to catch and it has been around for centuries. (R93)*

Some responses indicated that horse owners are making informed choices in regard to the permit and the appropriateness of vaccination in their situation.

*Not recommended for PPID [Pituitary Pars intermedia Dysfunction – Cushing’s disease] cases. (R104)*

*One of my horses does not meet the vaccination criteria as stated on the minor use permit. (R112)*

There were some comments (*n* = 16) that raised the issue of a Hendra virus area and vaccination not being necessary due to their geographical location relative the Hendra virus or flying-foxes.

*I don’t live in a Hendra area. I won’t put my horses at risk of an on-trial only vac...(*R28)

*Willing to take the risk as not in high flying fox area. (R06)*

*Extremely low flying fox/bat population in our area and no contact with horses from neighbouring properties. (R34)*

#### Theme 3: Attitude to authorities

A general sense of anger and resentment towards authorities, including veterinarians and pharmaceutical representatives, was evident in 36 comments from different individuals.

*The fact people are trying to force us to vaccinate. Vets are refusing to treat horses that are unvaccinated; some grounds will not allow horses that are unvaccinated /the amount of things being swept under the rug and the lies being toted around by the Vaccination Company and vets that are reading from the same song sheet.* (R17)

*We have chosen not to due to the way it has been handled and the lack of truthful information.* (R29)

Most of these comments indicate that horse owners connected veterinarians and the vaccine manufacturer as working together. This is evident when respondents comment on feeling forced into vaccination and bullied.

*I also will not be bullied into it by the government or vets making it mandatory. (R14)*

*The drug company is all about money and is pushing the vets’ hands so they won’t come out to unvaccinated horses. (R64)*

*I do not support the bullying and scaremongering tactics of vets and [vaccine manufacturer] to push this vaccine! (R59)*

Respondents were angered by their perception that veterinarians were using their professional power to force people to vaccinate by withholding their services if the horses were unvaccinated.

*Being Forced to choose a vaccine that is Unproven to work in order to gain veterinary care. (R148)*

*DISGUSTED at vets refusing to attend unvaccinated horse unless to vaccinate or to put to sleep. (R115)*

*I think the vets have over reacted to the extreme which also makes me so angry that I would rather shoot my horse than give them money to come out and refuse treatment. (R108)*

Respondents identify a lack of transparency in how their complaints and concerns were dealt with as a driver for not adopting vaccinations.

*Despite the attempts to cover up the amount of horses that have died following vaccination and others that have been rendered unrideable, not to mention the adverse reactions. The truth is the truth and this vaccine in its current form is not safe for horses in its current form. Veterinarians are not reporting all reactions and are using it for a cash cow and forcing it upon people who live in low risk areas. (R81)*

*During the investigation into the death of my horse [vaccine manufacturer] rep attempted to intimidate me, tried to guilt trip me and lied many times which he was caught out doing. (R45)*

*Reactions which [vaccine manufacturer] decides if it is a reaction or not. (R136)*

Overall, the comments from some horse owners when asked why they were electing not to vaccinate their horses reflect a lack of trust in the pharmaceutical company and veterinarians.

*Something here is NOT right! Too many cover ups in regards to reactions & deaths from the vaccine itself. (148)*

*As before it has been pushed onto the market with very poor research regardless of the spin they try to put on it. Too many reactions are not acknowledged and reports just disappear. I am not anti-vaccine of any kind. But I am strongly anti Hendra vaccine. (R126)*

*The fact that there is SO MUCH MISINFORMATION propagated by [vaccine manufacturer] who do not report the facts on reactions (but all horse owners know them) and vets who have a vested interest in pushing the vaccine for kickbacks is disgusting. (R114)*

### Factors influencing the potential uptake of vaccination

**Fig. 1:**
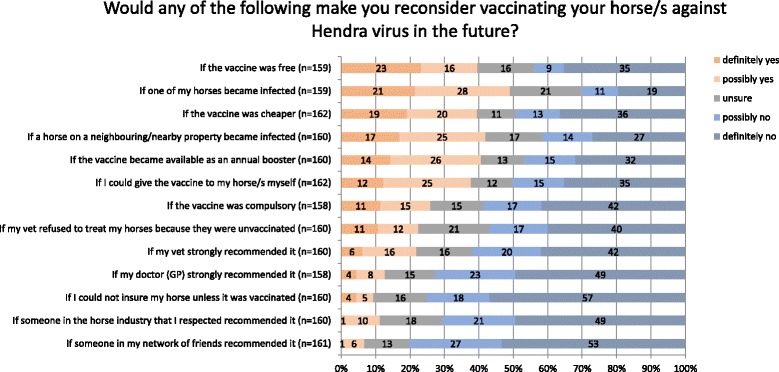
Factors that would influence uptake of Hendra virus vaccination. Data has been rounded

Respondents were asked to consider a series of situations and indicate whether the situations would cause them to reconsider vaccinating their horses against Hendra virus. Figure 1 summarises these findings.

In the top six influencing factors, 37 to 49% of respondents would reconsider vaccinating their horse(s). Issues of cost, convenience, immediacy of Hendra virus risk and personal control were features that linked more strongly to reconsideration of vaccination. Policy issues were reported to be less influential, with 23 to 26% of respondents saying they would reconsider vaccination in response to policy changes which made the vaccination compulsory, or veterinary policy changes to refuse treatment to unvaccinated horses. Recommendations from veterinarians and medical doctors were next down the list of self-reported influencers with 12- 22% saying they would reconsider vaccination following advice from them. The factors reported to be least likely to influence vaccination decisions were requirements for insurance (9%) and recommendations by others in the industry and friends (7 – 11%).

## Discussion

The single most frequent reason given for non-adoption of vaccination was concern about side effects from the vaccine. Many respondents felt that the risk of side effects from the vaccine outweighed the perceived risk of Hendra virus infection. Lack of trust and confidence in veterinarians and the pharmaceutical company also featured prominently in comments as drivers for the non-adoption of the vaccine for horses. Comments suggested that the perceived lack of testing of the vaccine, and feeling that their horses were being used as ‘guinea pigs’ also resulted in an unwillingness to vaccinate their horses against Hendra virus. Responses regarding side effects that were thought to be inappropriately acknowledged by the pharmaceutical company also figured significantly. The results will be discussed in the context of the themes identified.

### Barriers to uptake of vaccination

#### Theme 1: Attitude to the vaccine

Historically, attempts to claim that a technology is free from risk have sometimes resulted in a perception of increased likelihood of that risk [44, 45]. This may increase the lack of trust felt by those on the receiving end of the technology [46]. At the time of this study the discourse surrounding Hendra virus and the vaccine was further complicated because the vaccine was still being used under a restricted permit. This was perceived by some horse owners as evidence that there was something wrong with the vaccine and that adequate pre-release trials had not been carried out. Subsequent to our study data being collected, the vaccine became fully registered in August 2015 [47]. A summary of adverse experience reports by the Australian Pesticides and Veterinary Medicines Authority (APVMA), the agency responsible for assessment and registration of the vaccine, reported the adverse reaction rate at 0.001% in an estimated 100,000 horses vaccinated [48, 49] may not be sufficient to rebuild trust by horse owners that their concerns are being heard.

Some comments highlight the sense that the concerns of the horse owners are not being addressed or even acknowledged. Discounting by salaried experts of views that differ from the accepted scientific approach, or expressions of uncertainty around the risk of Hendra virus and the vaccine, may limit the opportunity for frank and open discussion. Acknowledgement of such expressions might enhance direct and transparent discourse around Hendra virus [50, 51] and continue into improved acceptance of recommended strategies. Open knowledge, or uncertainty sharing, could play a role in including horse owners as participants or stakeholders in the communication process and may help in the attenuation of risk perception surrounding Hendra virus and the vaccine, and enhance trust within stakeholder relationships [52]. Such uncertainty within scientific research is generally well considered by stakeholders and allows for resources to be invested in risk management strategies that focus on what is being done to reduce the uncertainty [53].

#### Theme 2: Risk assessment

Lay risk assessment is often considered to be based on ignorance or originating from faulty mental processing in regards to scientific information [10, 54]. Instead, the data collected provides evidence of careful consideration and a scientific approach to biosecurity. Some respondents cited the vaccine permit and the restrictions placed on vaccinating sick or immunocompromised horses [47] as their reason for not vaccinating. If this response is considered as noncompliant, then it does appear to make nonsense of the permit and the restrictions that are in place around the vaccine use.

Comments that indicated respondents were undertaking a personal risk assessment based on identifying areas as being more or less likely to have a Hendra virus case are of concern. Research has identified that while some areas have had more Hendra virus cases historically, given the flying-foxes’ spatiotemporal distribution across Australia, it is difficult to predict where cases might occur [27]. The possibility of direct transmission from flying-fox urine to horses also suggests that even small numbers of flying-foxes may dramatically increase the risk of an infection in horses [22, 24]. Therefore, it is important to address the uncertainty surrounding Hendra virus cases and ensure that all stakeholders are fully aware of the changing nature of flying-fox distribution, and consequently risk of infection with Hendra virus, over time and space.

The comments suggesting that Hendra virus has been around for a long time are accurate [55] and should not be dismissed. However, the connection made by respondents between the historical nature of the virus and the lack of need for a vaccine would benefit from further consideration.

#### Theme 3: Attitude to authorities

The role of veterinarians in the provision of information and communication around animal welfare and health, and their influence on horse owner behaviour is even more crucial in the context of Hendra virus, given the potential for human infection and its high morbidity and mortality in horses and people. This reinforces the urgent need to ensure that the veterinarian-owner relationship is protected and supported in the current Hendra virus discourse. A breakdown in trust and credibility could have long-lasting and far-reaching consequences in any future emergency animal disease response [46].

There was a strong perception amongst some respondents of a conspiracy between the pharmaceutical company that manufactures the vaccine and veterinarians who must administer it and hence benefit financially. When information and recommendations are provided by sources that have commercial interests in the uptake of such information, the reliability of such material may be doubted [56]. Such complications surrounding the release of the Hendra virus vaccine communication should have been anticipated [46].

Some respondents’ comments reflect an egalitarian worldview, one in which value is placed on a society free from stratification about who can offer instruction and direction [57]. This is in contrast with the traditional hierarchical worldview on which science is founded. Consideration by veterinarians of the role that worldview plays in their approach to risk, as well as in their clients’ decision making may assist in creating opportunities for more open and participatory communication.

As previously discussed, the Hendra virus vaccine was initially released under a restricted permit. This meant that all Hendra virus vaccine marketing and advertising, as well as administration, could only be carried out by veterinarians, further cementing the connection between the vaccination and the profits of those administering it. Other horse vaccines in Australia can be purchased from a veterinarian by their regular clients and administered to the horse by their owner. It perhaps should have been anticipated that a dramatic change in vaccine administration would cause concern and suspicion amongst horse owners within the climate of uncertainty surrounding an emerging zoonotic virus.

### Factors influencing the potential uptake of vaccination

In the final section of the study, factors that might influence horse owners to reconsider vaccinating their horses in the future were considered. A first hand or near miss experience with Hendra virus infection was most commonly reported as likely to influence potential adoption of vaccination. While this is useful and hardly surprising data, it does not inform changes in communication policy or appear to offer practical changes that can be implemented by horse owners or veterinarians, except to wait for the next infection. This phenomenon is however, supported by anecdotal reports from veterinarians in areas where there have been previous Hendra virus cases followed up a rapid increase in uptake of vaccination [58], and by peaks in laboratory submissions and visits to government Hendra virus websites following reported cases [59].

Reduction of cost was reported as a likely influencer of increased uptake, with free vaccine, cheaper vaccine, less frequent booster doses and owner-administration potentially increasing vaccine uptake. Similarly, the potential for government subsidy for the vaccine was viewed favourably. Since the vaccine’s release, incentives have been used to increase uptake of vaccination. These include free second vaccine doses offered by the vaccine manufacturer, and locally arranged events with ‘bulk vaccination’ to reduce veterinary costs. These appear to be effective and successful approaches, although no published data are available to verify this. On the other hand, to a sizable group of respondents in this research, these offers make no difference to their stated intended uptake and, in fact, may fuel further suspicions of pressure to vaccinate for profit driven or other sinister reasons.

The influence of veterinarians, medical doctors, respected others, and friends appeared less likely than other factors examined to influence decisions regarding the uptake of vaccination and this should not be surprising. Data from the equine influenza outbreak in Australia in 2007-08 identified the importance of veterinary and other horse health professionals’ infection control support in horse owner perceptions of outbreak management and effectiveness of biosecurity measures [60, 61].It is also clear that for the majority of respondents in this study, recommendations from health professionals and networks do not play a strong role in decisions around the uptake of Hendra virus vaccine. Research confirms that individuals (salaried experts and lay people alike) will align themselves with people that support their worldview while making decisions that support and maintain this identity [17]. Those who consider themselves as equally qualified with a salaried expert are less inclined to take advice from so-called “experts” [13].

This study provides an insight into the risk perception and practices of an important target audience for animal health agencies in Australia. In order to better assess its effectiveness, consideration of the limitations of the study should be undertaken. Given there is no large or reliable database of Australian horse owners from which to draw a representative sample, it is most common to adopt a self-selected approach to collect cross-sectoral industry data [29, 62]. The survey sample is also limited to those who access Facebook and either ‘like’ one of the participating veterinary clinic’s Facebook pages or have social networks that have these links. This approach allows access to horse owners with a level of connection to a veterinary clinic in a target region. However, it does not limit the sample only to those who are registered with one of these practices. In fact, cross posting could be traced to various side groups including a dressage club.

Another pertinent issue is that given the current climate of the discourse around Hendra virus and vaccination in horses, it is possible that respondents may have held certain biases and had different motives to respond (or not) to the survey. Those who chose not to respond may have considered the survey part of a ‘push’ for vaccination, and those who did respond may have felt it was an opportunity to vent frustrations. Although issues of responder bias cannot be dismissed the data collected appear to be comparable to other similar studies in terms of sample demographics [29]. The deliberate selection of those who have not vaccinated would be expected to attract respondents with more negative and/or less trusting, and more questioning views of vaccination and possibly government agencies and other stakeholders. Such views are of direct relevance to the study and are therefore not viewed as a study weakness.

## Conclusion

Horse owners in Australia are encouraged to vaccinate their horses against Hendra virus and to adopt a number of on-property risk mitigation practices. Both pharmaceutical and property management strategies are aimed at reducing the probability of infection from flying-foxes of horses on pasture. This research identifies self-reported reasons for the rejection of Hendra virus vaccination and seeks to identify perspectives that inform better communication and policy that may encourage or promote uptake of the vaccine.

Increased vaccination uptake could be encouraged by reducing vaccination costs and lowering concerns about the vaccine’s safety and effectiveness. The latter approach is challenging; more time and data may be required to convince a sceptical or distrusting audience. The qualitative and quantitative evidence that veterinarians are not trusted within this discourse is of concern. The need to rebuild and protect good relationships between horse owners and veterinarians should be highlighted as a priority issue, as veterinarians have the most direct and frequent contact with owners, particularly during disease outbreaks. Once lost, trust in veterinarians will be hard to win back.

Tasked with improving the general understanding of horse owners’ risk perception and their responses to Hendra virus risk, this research highlights the fundamental paradigm shift that is required for policy makers, veterinarians, and researchers to fully understand and participate in the Hendra virus discourse. Such a discourse requires scientific facts but also needs, perhaps more urgently, a reconsideration of how risk perception, trust and decision making are considered.

Qualitative analysis of these data illustrated the complex nature of decision making around risk and the adoption of protective behaviours. Previous research into risk perception generally and zoonotic disease management specifically has highlighted the importance of avoiding the assumption that any one research approach is unambiguous or empirically pure [18, 35]. Rather, as relationships and identities are dynamic, incomplete and always context specific, so too values, beliefs and responses can change as meaning is made [1]. Beliefs cannot be captured as discrete, measurable and static entities. Adoption of traditional quantitative research approaches alone, while able to report on the ‘who is doing what’, may obscure the ‘how’ and ‘why’. Within such a discourse, the inherent assumptions on which quantitative research is based can render the more useful and significant social aspects of the risk discourse invisible. This often results in development of programs and risk management approaches that misfire, inflame the discourse and fail. Considering decisions around risk as being based solely on logic and information is misguided. Likewise, labelling noncompliant behaviour as irrational and faulty and subsequently disqualifying it from the discourse is unhelpful and counterproductive. Instead, by understanding that salaried experts and lay people alike make decisions in disease risk situations based on mental and affective shortcuts, founded on how they view the world and who should have control and make decisions could increase mutual understanding of the multitude of factors involved in these complex decisions. Promotion of transparent discussion with all stakeholders, including those actually at risk can result in a more robust and progressive dynamic discourse that is able to adapt within the changing face of an emerging infectious disease and responsive technologies that follow.

## Additional file

Additional file 1: Horse owners and Hendra virus on-line survey. Description: Survey with all skip logic removed. (PDF 405 kb)
