# Supplementary material for: “Why won’t they just vaccinate?” Horse owner risk perception and uptake of the Hendra virus vaccine
Source: BMC Vet Res. 2017 Apr 13;13:103. doi: 10.1186/s12917-017-1006-7 (PMC5390447; doi:10.1186/s12917-017-1006-7)
Supplement: Additional file 1: — Horse owners and Hendra virus on-line survey. Description: Survey with all skip logic removed. (PDF 405 kb) [file 12917_2017_1006_MOESM1_ESM.pdf]

Introduction

STUDY INFORMATION: This survey forms part of a research project looking at Hendra virus risk management attitudes and practices of horse owners who have non-vaccinated horses. This research, and the information you provide, will be of great value to those working in animal disease policy and response, as well as veterinarians and public health professionals. This project is separate from but will complement the recently completed HHALTER project.

FUNDING: This survey is being conducted by the University of Western Sydney, and is being supported by the State of Queensland, the State of New South Wales and the Commonwealth of Australia under the National Hendra Virus Research Program.

STRUCTURE: The survey has been designed to be as quick and easy to answer as possible. Most questions are multiple choice, and you can write comments and give additional opinions if you WANT to. Some questions are skipped over - depending on the responses you give – most are marked with an asterisk (*) to indicate a response is needed to be able to move on.

TIME: The survey will probably take around 20-30 minutes to complete.

EXITING/RE-ENTERING: We would generally recommend that you try to complete the survey in a single session, however, the survey is set-up so that you can partially complete it, exit, and go back in as often as you need to. Once you complete the survey and press the 'done' button at the end you cannot change responses. Occasionally problems are encountered if your computer is set on high security settings. If you have problems please see the note below and/or contact the researchers if you need more assistance.

CONFIDENTIALITY: The data you provide will be treated in confidence and only combined data will be reported. No individual data will be reported and no identifiable data will be made available to anyone.

CONTACT: If you have any questions about this study please e-mail Dr Mel Taylor at the University of Western Sydney (melanie.taylor@uws.edu.au).

IF YOU ARE WILLING TO TAKE PART IN THIS SURVEY PLEASE CLICK ON THE “NEXT” BUTTON BELOW WHEN YOU'RE READY TO BEGIN.

Background Notes

ETHICS: This study has been approved by the University of Western Sydney Human Research Ethics Committee. The Approval Number is H10643. If you have any complaints or reservations about the ethical conduct of this research, you may contact the Ethics Committee through the Office of Research Services on Tel 02 4736 0013 or email humanethics@uws.edu.au. Any issues you raise will be treated in confidence and investigated fully, and you will be informed of the outcome.

HIGH SECURITY SETTINGS: Ensure you have 'cookies enabled' BEFORE starting the survey. The survey host website uses cookies to recognise your response and this allows you to exit/return/edit responses. You need to have 'cookies enabled' in your browser for this to work. The vast majority of people will have no problem. However, if you're unsure about your settings or are worried about losing data we suggest you complete Question 1 (on Page 2), click NEXT (to save Page 2 data) and then use EXIT EARLY on Page 3 to leave the survey - if there is going to be a problem this will show up now. If everything is OK you can then re-enter the survey (using your original link) - this should open up on Page 3. You can then go back to complete the Page 2 questions - or adjust your browser settings if you need to.

Hendra virus vaccination

*1. Have you vaccinated your horses against Hendra Virus?

- ○ Yes, all of them
- ○ Yes, some of them
- ○ I have vaccinated some/all of them but have stopped vaccinating some/all of them
- ○ No, none of them

You and your horses

These questions focus on the horse/s you own or have responsibility for, and information about you and your horse property. This section provides really useful background information that is important for the study and for analysis of the data.

2. How many horses do you have that you...

own/part own?

lease?

manage?

3. Are you someone who makes, or influences, decisions regarding...

	Yes	No
your horse/s' health? (e.g. whether to vaccinate)		
the management of your horse property? (e.g. fencing, location of food/water)		

Comment (if required)

4. Are your horse/s located in an area where a Hendra virus case in horses has previously been reported?

- Yes - within 20km of a Hendra virus case in horses
- Yes - within 20-50km of a Hendra virus case in horses
- Within 50-100km of a Hendra virus case in horses
- No - more than 100km from a Hendra virus case in horses
- Don't know

5. Do you...

	Yes	No	Unsure
...ever see flying foxes flying over your horse property?			
...ever see flying foxes in trees on your horse property (e.g. eating, roosting)?			
...have any fig or mango trees on your horse property?			

6. What is the postcode of the property where your horses are kept? (just the four numbers)

postcode (number only)

7. Do you earn income from a horse related business or activity?

- ○ Yes, as my MAIN source of income
- ○ Yes, as an additional (secondary) source of income
- ○ No

Comment

8. What is the nature of your involvement with horses? (Tick all those that apply)

- ☐ Recreational (eg riding for pleasure, Riding for the Disabled, Australian light horse)
- ☐ Thoroughbred Racing
- ☐ Harness Racing
- ☐ Competitive riding / Equestrian (eg campdraft, carriage driving, cutting, dressage, endurance riding, eventing, hacking, jumping, polo, polocrosse, pony club, reining, showing, vaulting, western, working equitation)
- ☐ Breeding
- ☐ Stabling/Agistment
- ☐ Commercial (eg riding school, horse tourism, animal-assisted therapy)
- ☐ Working/Farming/Stock horses
- ☐ Other

Please specify or provide details

9. Do the following people come onto your horse property and have contact with your horse/s?

	Yes	No
Adults (outside your immediate household)	○	○
Employees	○	○
Children (your own, or other people's)	○	○

10. Have you been affected or impacted by a previous Hendra Virus case (either directly or indirectly)?

- ○ Yes
- ○ No

If you feel you can, please provide a little information.

11. Are you...

- Male
- Female
- Rather not say

12. How old are you?

- under 35
- 35-44
- 45-54
- 55-64
- 65 or over
- Rather not say

General horse health

These questions are about the general health of your horse/s, contact with your veterinarian, and any health concerns that you might have for your horses.

13. In the past 12 months, how many times...

	0	1-2	3-4	4+
... have you spoken to a veterinarian about your horse/s? (total number of conversations)	○	○	○	○
... has a veterinarian examined your horse/s? (total number of examinations/consults)	○	○	○	○

14. In the last 12 months, have any of your horses been vaccinated for...?

	yes - all of them	yes - some of them	no
Tetanus	○	○	○
Strangles	○	○	○
Other	○	○	○

Please provide details of other vaccination(s)

15. If you are choosing to vaccinate some of your horses and not others, can you tell us how you decide which ones to vaccinate/not vaccinate?

16. Please consider the following four health concerns. How would you rank them for your horses currently - from 1st (of greatest concern to you) to 4th (of least concern to you).

	1st - greatest concern	2nd	3rd	4th - least concern
Colic	○	○	○	○
Laminitis	○	○	○	○
Hendra virus	○	○	○	○
Traumatic accident	○	○	○	○

Is there any other health concern you would have included above? (please give details)

17. OPTIONAL: Do you have any comments you would like to make regarding your horse/s' health?

Assessing your property for Hendra virus risk

18. Looking around your horse property, have you thought about the risk of Hendra virus infection in your horses and made any property changes?

Please select one option that best describes your approach.

- I have not assessed the property for the risk of Hendra virus infection
- I have assessed the property, I don't think there is a risk, and I have not made any changes
- I have assessed the property, I think there is a risk, I intend to make changes, but I have not done so yet
- I have assessed the property, I think there is a risk, but I have decided there is nothing/nothing more I can do to reduce the risk
- I have assessed the property, I think there is a risk, and I have made changes to reduce the risk

Comments/Is there are better way to describe your situation?

Property management strategies to reduce Hendra virus risk

This next section asks about your use of some property management strategies that could reduce the risk of Hendra virus - and your views on each.

You will be asked about
- covering horses' food bins/containers - where horse/s are hand-fed
- covering horses' water containers - where water is provided in this way
- keeping horses off pasture when flying foxes are active
- keeping horses away from fruiting/flowering trees.

19. How EFFECTIVE do you think the following are/would be at reducing the risk of Hendra virus infection in your horse/s?

	Not at all	A little	Moderately	Very	Extremely
Covering horses' food bins/containers	○	○	○	○	○
Covering horses' water containers	○	○	○	○	○
Keeping horses off pasture when flying foxes are active	○	○	○	○	○
Keeping horses away from fruiting/flowering trees	○	○	○	○	○

20. How EASY was it/would it be for you to do the following to reduce the risk of Hendra virus infection in your horse/s?

	Not at all	A little	Moderately	Very	Extremely
Covering horses' food bins/containers	○	○	○	○	○
Covering horses' water containers	○	○	○	○	○
Keeping horses off pasture when flying foxes are active	○	○	○	○	○
Keeping horses away from fruiting/flowering trees	○	○	○	○	○

We're now going to ask you briefly about each of the property management strategies. Starting with covering horses' food containers

*21. Are you covering your horses' food containers?

○ Yes for all my horses

○ Yes for some of my horses

○ No

○ Not applicable - I don't hand-feed my horses / there are no food containers to cover

Comments

Covering horses' food bins/containers

22. To what extent have the following factors influenced your decision NOT to cover all/some of your horses' food bins/containers?

	Not at all	A little	Somewhat	A lot	A great deal
the time it would take to do	○	○	○	○	○
the money it would cost to do	○	○	○	○	○
the practicalities of doing it	○	○	○	○	○

Other factors involved in your decision not to cover your horses' food bins/containers

Covering horses' water containers

*23. Are you covering your horses' water?

- ○ Yes for all my horses.
- ○ Yes for some of my horses.
- ○ No
- ○ Not applicable - my horse/s only have access to water in a dam/creek/large water source

Covering horses' water containers

24. To what extent have the following factors influenced your decision NOT to cover all/some of your horses' water containers?

	Not at all	A little	Somewhat	A lot	A great deal
the time it would take to do	○	○	○	○	○
the money it would cost to do	○	○	○	○	○
the practicalities of doing it	○	○	○	○	○

Other factors involved in your decision not to cover your horses' water containers

Keeping horses off pasture when flying foxes are active

***25. Do you keep your horses off pasture when flying foxes are active, eg. dawn and dusk?**

- Yes, all my horses
- Yes, some of my horses
- No
- Not applicable - there aren't any flying foxes active around my horse property

Keeping horses off pasture when flying foxes are active

26. To what extent have the following factors influenced your decision NOT to keep your horses off pasture when flying foxes are active?

	Not at all	A little	Somewhat	A lot	A great deal
the time it would take to do	○	○	○	○	○
the money it would cost to do (e.g. space, facilities)	○	○	○	○	○
the practicalities of doing it	○	○	○	○	○

Other factors involved in your decision not to keep your horses off pasture when flying foxes are active

Keeping horses away from fruiting/flowering trees

*27. Do you keep your horses away from fruiting/flowering trees?

- Yes, all my horses
- Yes, some of my horses
- No
- Not applicable - I don't have any fruiting/flowering trees on my horse property

Keeping horses away from flowering/fruiting trees

28. To what extent have the following factors influenced your decision NOT to keep your horses away from flowering/fruiting trees?

	Not at all	A little	Somewhat	A lot	A great deal
the time it would take to do	○	○	○	○	○
the money it would cost to do (e.g. fencing)	○	○	○	○	○
the practicalities of doing it	○	○	○	○	○

Other factors involved in your decision not to keep your horses away from fruiting/flowering trees

Supporting use of Hendra virus risk reduction strategies (including vaccina...

In this section we want to find out what might encourage you to use some of the property management strategies to reduce Hendra virus risk that you haven't taken up (those strategies covered in the previous set of questions) and what you think could be done to encourage use of those strategies (and vaccination) by other horse owners.

29. Would you consider taking up some/some more of the recommended property management strategies...

	Definitely no	Probably no	Unsure	Probably yes	Definitely yes
if one of your horses became infected with Hendra virus?	○	○	○	○	○
if your friends adopted some of those strategies?	○	○	○	○	○
if a neighbouring/nearby property had a Hendra virus case?	○	○	○	○	○
if people you respect recommend you adopt some of those strategies?	○	○	○	○	○
if a professional (veterinarian or consultant) conducted a risk assessment and recommended you adopted some of those strategies?	○	○	○	○	○
if government funding was provided to assist?	○	○	○	○	○

Other situations when you would consider taking up property management strategies (please specify)

30. If you were responsible for encouraging the adoption of risk management strategies by horse owners against Hendra virus infection in their horses, what approaches would you consider using?

Please select as many options as you like - and add any other suggestions for things you think might be effective.

- ☐ Posting/emailing pamphlets about property management and vaccination
- ☐ Holding local information nights
- ☐ Using social media to provide information
- ☐ Arranging for subsidised (reduced cost) vaccinations
- ☐ Offering subsidised (reduced cost) professional risk assessments
- ☐ Making vaccination compulsory in places/regions where there have been identified cases

What other approaches would you consider taking?

Your views on Hendra virus risk

These questions ask for your general views on Hendra virus risk and its consequences

31. How LIKELY do you think it is it that a Hendra virus case could occur...

	Not at all	A little	Moderately	Very	Extremely
in one of your horses?	◯	◯	◯	◯	◯
in a neighbour's horse or on a horse property near by?	◯	◯	◯	◯	◯
in a friend's horse?	◯	◯	◯	◯	◯
in your area?	◯	◯	◯	◯	◯

Comments

32. How SERIOUS would it be if there was a Hendra Virus infection...

	Not at all	A little	Moderately	Very	Extremely
in one of your horses?	◯	◯	◯	◯	◯
in a neighbour's horse or on a horse property near by?	◯	◯	◯	◯	◯
in a friend's horse?	◯	◯	◯	◯	◯
in your area?	◯	◯	◯	◯	◯

Comments

33. Please indicate the extent to which you disagree or agree with the following statements.

	Strongly disagree	Disagree	Neither agree or disagree	Agree	Strongly agree
I want to try and do everything I can to prevent my horses catching Hendra virus.	○	○	○	○	○
There is no point vaccinating against Hendra virus because there are still other unvaccinated horses.	○	○	○	○	○
I avoid thinking about the possibility of Hendra virus infection in my horses.	○	○	○	○	○
If my horses are going to get Hendra virus, there is little I can do about it.	○	○	○	○	○
Nothing I do will make a difference to the risk of my horse catching Hendra virus.	○	○	○	○	○
If my horse catches Hendra virus, it will not be the worst thing in the world.	○	○	○	○	○
I want to learn more about the connection between Hendra virus and strategies designed to prevent Hendra virus infection.	○	○	○	○	○
I can protect my horses from Hendra virus without help from vets and the government.	○	○	○	○	○
I have assessed the risk of Hendra virus in my horse and my actions provide enough protection.	○	○	○	○	○

Comments

34. What three (single) words come to mind when you think about Hendra Virus?

1.

2.

3.

Your views on Hendra virus vaccination

Our study is designed to focus on horse owners who have not vaccinated their horses (and including those who have stopped vaccinating), to investigate their use of other risk reduction strategies and their attitudes towards Hendra virus risk generally.
Although vaccination is not the focus of the study, in this last section we would like to know a little bit about your attitudes to Hendra virus vaccination.

35. Just generally, what are your reasons for not vaccinating your horse/s against Hendra virus (or for withdrawing them from vaccination, if applicable)?

36. Would any of the following make you re-consider vaccinating your horse/s against Hendra virus in the future?

	definitely no	possibly no	unsure	possibly yes	definitely yes
If a horse on a neighbouring/nearby property became infected	○	○	○	○	○
If my vet strongly recommended it	○	○	○	○	○
If the vaccine was compulsory	○	○	○	○	○
If my doctor (GP) strongly recommended it	○	○	○	○	○
If I could give the vaccine to my horse/s myself	○	○	○	○	○
If I could not insure my horse unless it was vaccinated	○	○	○	○	○
If one of my horses became infected	○	○	○	○	○
If someone in my network of friends recommended it	○	○	○	○	○
If my vet refused to treat my horses because they were unvaccinated	○	○	○	○	○
If the vaccine was cheaper	○	○	○	○	○
If the vaccine became available as an annual booster	○	○	○	○	○
If someone in the horse industry that I respected recommended it	○	○	○	○	○
If the vaccine was free	○	○	○	○	○

What other things might make you consider vaccinating your horse/s against Hendra Virus?

Final Comments

37. OPTIONAL: If you have any comments you'd like to make please write them in the space below.

REQUEST FOR FOLLOW UP: This survey is the first part of our research project. We are looking for a small number of people who might be willing to take part in an interview with one of the research team. The interview would be held locally, at the time and place of your choosing, and would take around 40-60 minutes.
For taking part in the interview,to thank you for your time, you will receive a $30 gift voucher.

If you would be willing to consider speaking with one of us, please provide some contact details below.

Reassurance: Your details will NOT be passed on to anyone or reported as any part of this project, and you will not receive any marketing or SPAM/junk e-mails. You will only be contacted by researchers in this project and your future participation would be entirely voluntary.

Thank you for any further assistance you might be willing to give.

38. CONTACT DETAILS (if willing to be contacted about an interview)

Your name (first name)

Contact e-mail address

Phone number
